# An explainable machine learning framework for cardiovascular risk prediction using structured health data

**DOI:** 10.3389/frai.2026.1812622

**Published:** 2026-04-29

**Authors:** Valeru Vision Paul, Jafar Ali Ibrahim Syed Masood

**Affiliations:** 1 School of Computer Science and Engineering, Vellore Institute of Technology, Vellore, Tamil Nadu, India; 2 Department of Quantum AI, School of Computer Science and Engineering, Vellore Institute of Technology, Vellore, Tamil Nadu, India

**Keywords:** cardiovascular risk prediction, explainable artificial intelligence, interpretable models, machine learning, SHAP analysis

## Abstract

**Background:**

Heart disease (CVD) is still one of the leading causes of death worldwide. As a result of complex clinical data, more common applications of machine learning models for CVD risk prediction. Yet, many machine learning methods suffer from a lack of interpretability which will make it hard for them to be employed in the clinical setting. This study introduces an interpretable ML framework for predicting cardiovascular risk using structured clinical data.

**Methods:**

This study used a publicly available cardiovascular dataset consisting of about 70,000 patient records. It contains various demographic, physiological, and lifestyle-related variables normally utilized in cardiovascular risk evaluation. For five folds, Stratified Cross Validation was performed to develop three ML models, namely LogisticRegression(), RandomForestClassifier(), and GradientBoosting Classifier(). The model performance at various evaluation metrics, such as accuracy, precision, recall, F1-score and area under the receiver operating characteristic curve (AUC-ROC) were measured. SHAP (Shapley Additive Explanations) was used to explain both global and local feature contributions in an effort to improve interpretability.

**Results:**

The models evaluated for the experimental results displayed similar prediction performance with ensemble-based methods performing better. Voting Ensemble model was scored second with an AUC of 0.793 (Gradient Boosting had the highest predictive performance: 0.794). The models achieved an AUC well above the baseline Logistic Regression model performing at 0.773. The higher accuracy of ensemble models is mainly due to their ability to capture nonlinear interactions between features in the dataset.

**Discussion:**

In terms of the most influential predictors across all models, the explainability analysis found that age, blood pressure, cholesterol levels and weight were predominantly* included. By building on the application of explainable artificial intelligence techniques with machine learning models, these results show how such approaches can lead to more transparent and interpretable cardiovascular risk prediction. This framework demonstrates the potential of explainable machine learning to facilitate clinical decision-making and build trust in predictive healthcare models.

## 1 Introduction

### 1.1 Artificial intelligence and the need for transparent predictive models

These days, artificial intelligence is changing how researchers handle organized health and behavior information. Instead of relying only on traditional statistics, scientists now turn to machine learning to spot hidden trends in large datasets - sometimes predicting outcomes older methods miss. Still, as models grow more complicated, understanding their choices gets harder. According to Lehr, strong prediction power alone is not enough; meaning comes from knowing why a model decides what it does - and whether those decisions hold up under scrutiny.

Despite varied research in Explainable AI, one point stands clear - understanding model decisions helps people rely on them. Instead of treating algorithms like hidden mechanisms, techniques aim to reveal which inputs shaped outputs, supporting clarity through transparency. When applied to health settings, where overlapping risk factors often appear together, dependable systems become essential for responsible practice.

### 1.2 Cardiovascular: risk modeling as a structured data challenge

Heart troubles continue drawing attention in data-focused medical studies, mainly because patterns within patient details are intricate yet plentiful. Because information like age, blood pressure, daily habits feed forecasting tools, heart datasets fit well when testing machine learning methods. Lately, scientists explored diverse ways to anticipate cardiac issues, pulling techniques from both straight- line calculations and more flexible, curved-pattern systems.

However, this work is heavily biased toward prediction quality; little or no analysis of how models utilize features internally has been discussed. In other words, many times model fit indices are calculated without testing for dependence of the models on stable or invariant explanations. This gap restricts the extent to which we can interpret and glean methodological insight from comparative machine learning studies (Lu et al., 2021).

### 1.3 Explainable artificial intelligence and SHAP-based interpretation

Explainability techniques are key for addressing the transparency challenge in machine learning. Among them, SHapley Additive exPlanations (SHAP) is a theoretically motivated approach based on cooperative game theory to decompose the prediction from other features. SHAP admits global interpretation through feature importance summary at a system level, as well as local interpretation by per-prediction explanation (Chari et al., 2021; Rasheed et al., 2022; Hildt, 2025).

SHAP has been broadly used in healthcare machine learning research, but primarily as a visualization tool without the explicit analysis to compare model behavior. As a result, little is known about how explainability patterns evolve across a variety of model architectures. Investigating such variation is important for examining whether interpretability findings are generalizable or model-specific.

Recent advances in explainable machine learning have further improved cardiovascular disease prediction by combining ensemble learning with feature engineering and model interpretation techniques. For example, Ghose et al. (2022c). proposed a stacked ensemble framework that integrates multiple feature selection methods with SHAP-based explanations to enhance diagnostic performance and interpretability in heart disease prediction. While such approaches demonstrate strong predictive performance, most studies emphasize accuracy improvements through complex ensemble architectures while offering limited investigation of explanation stability across different model families. Furthermore, interpretability analyses are often conducted on a single model architecture, leaving open questions regarding whether identified risk factors remain consistent across diverse learning algorithms. Addressing this limitation, the present work focuses not only on predictive performance but also on the consistency of explainability across linear and ensemble models, enabling a more robust evaluation of interpretable machine learning for cardiovascular risk prediction.

To address this limitation, the present study examines both predictive performance and the consistency of explainability across linear and ensemble models. This approach enables a more robust evaluation of interpretable machine learning methods for cardiovascular risk prediction.

Recent state-of-the-art (SOTA) studies have investigated hybrid machine learning frameworks and explainable artificial intelligence techniques for cardiovascular disease prediction. For instance, recent research has introduced explainable ensemble learning architectures that integrate advanced feature selection with SHAP-based interpretation to enhance predictive accuracy in cardiovascular datasets. Additionally, hybrid machine learning frameworks that incorporate multiple classifiers have demonstrated strong performance in healthcare prediction tasks (Ghose et al., 2025; Ghose and Jamil, 2026). Although these methods achieve high predictive accuracy, they often depend on complex hybrid architectures and do not systematically examine the stability of feature explanations across different model families. In contrast, the present study assesses the consistency of explainability across Logistic Regression, Random Forest, and Gradient Boosting models using SHAP-based interpretation, thereby offering further insights into the robustness of feature contributions in cardiovascular risk prediction.

### 1.4 Dataset integration and methodological motivation

Curated benchmarking sets are readily available for rigorous evaluation of machine learning approaches. The dataset we introduce in this work integrates information coming from different well-known sources, such as the Cardio dataset and cardiovascular datasets available on the UCI Machine Learning Repository, into a heterogeneous benchmark that is meant to be used for comparative purposes. This integration allows us to investigate feature behavior on a wide variety of patient profiles while preserving standardized experimental settings.

We make no such claims in this paper about proposing a new predictive method. Second, the methodological emphasis is on how varied model families encode risk-related information and how explainability methods can uncover consensus or disagreement in feature correspondence. One way to assess models involves a less explored angle - comparing them through consistent explanations instead of relying solely on accuracy-driven distances.

### 1.5 Research objective and contributions

This study evaluates how different machine learning models provide interpretable explanations when predicting cardiovascular risk. Rather than simply ranking accuracy, attention turns toward transparency - using SHAP values to unpack decisions across three methods: Logistic Regression, Random Forest, and Gradient Boosting. Each model faces identical conditions, allowing comparison not just on predictions but on how well those predictions can be understood. The analysis therefore emphasizes interpretability in addition to predict performance.

- Examining how individual versus combined algorithms perform when applied to uniform heart health datasets. Different predictive approaches face off using structured medical inputs from varied origins. Performance shifts appear when stacking models instead of relying on one standalone method. Results depend heavily on data consistency across sources feeding into each technique. Single models sometimes hold their ground against more complex group setups.
- A fresh look at how consistently models justify their decisions, using SHAP both broadly and in specific cases. Different structures handle reasoning clarity in distinct ways. Where one model shows stable logic globally, it may falter when examined up close. Patterns emerge only when comparing multiple setups side by side. Some frameworks hold steady; others shift unpredictably between wide and narrow views. Clarity does not always transfer from overall trends to individual instances.
- This study Theerefore evaluate interpretability together with traditional performance metrics A different angle emerges when considering how explanations might stand alongside traditional metrics in assessing reliable artificial intelligence systems. What matters here is not just accuracy, but whether decisions can be made transparent through structured reasoning. Instead of treating clarity as an afterthought, it becomes a measurable factor in its own right. Performance gains new context once interpretability enters the evaluation framework. Trust shifts from being assumed toward something grounded in visible logic. A key challenge in clinical machine learning is achieving strong predictive performance while maintaining interpretability so that clinicians can understand the reasoning behind model predictions. Reliability grows stronger when users see not only outcomes, but also paths leading to them.
- By stressing interpretability stability rather than accuracy improvement only, this study provides evidence-based and transparent AI methods for the interpretation of health- related structured data-enabled parties.

This work highlights consistent understanding over mere precision gains, offering clear, tested approaches to artificial intelligence that support reliable reading of organized health information by involved groups.

### 1.6 The scientific importance of our study

Beyond predictive accuracy, interpretability of machine learning models is increasingly important in healthcare applications. Through this lens, clarity gains weight alongside traditional metrics. Examining shifts in feature influence across different learning methods opens doors within accountable AI inquiry. Instead of just asking how well a system works, we begin questioning how it reasons - revealing insights hidden beneath standard benchmarks. Explainability techniques enables deeper analysis of feature contribution and model reasoning, exposing nuances raw accuracy cannot reflect. Transparency grows not from claims but from observable behavior embedded in workflows. Reproducibility strengthens when processes expose their inner logic clearly. Rather than chasing ever-higher scores, future progress may hinge on valuing consistency, openness, and grounded interpretation. As expectations evolve, so must criteria guiding development - placing understanding above output. Stability matters - not only in results, but in reasoning pathways too.

## 2 Literature review

### 2.1 Machine learning in healthcare and prognostics

Over 10 years, machine learning’s push into spotting illnesses shifted sharply. That force found new ground in heart disease detection. Machine learning models are designed to identify patterns in clinical data and assist in prioritizing high-risk cases for early intervention (Aldahiri et al., 2021). Methods evolved to classify signals, guide early decisions. Work by Al-Alshaikh in 2020 marked part of this turn (Graham et al., 2021; Gnanavelu et al., 2025; Perveen et al., 2020).

The resilience of ensemble learning and gradient boosting to impose a non-linear risk on health has since been supported in various disease domains (Li et al., 2023). This transition from classical statistical methods toward machine learning algorithms introduces several advantages, the most important one being in a position to handle high-dimensional datasets and identify complex non-linear relationships that are frequently ignored by older methods (Lübeck et al., 2025; Ren et al., 2024; Upadhyaya et al., 2022; Yang et al., 2025).

### 2.2 Cardiovascular risk stratification with ML

Risk stratification earlier in time is now possible with ML on high-dimensional EHR and wearable data (Sarraju et al., 2021; Xu et al., 2020; Ogunpola et al., 2024). Sarraju et al. (2021) reported ML led-predictive model that performed better than the traditional risk scoring system. Nevertheless, one fundamental limitation of these studies is that they are conducted as a black-box analysis. Despite its superiority, the majority of ML models are black-box models and remain not generalizable to clinical practice (Sajid et al., 2022; Twick et al., 2022). SHAP LIME counterfactual reasoning have been suggested as an intuitive means to justify the ML decisions in clinical (Song et al., 2025; Ladbury et al., 2022) domain Increasingly it is becoming important to use machine learning models due to exponential rise in availability of real-world medical data via electronic health records (EHR) through which some of the physiological patterns and progression of a disease will get embedded but often ignored by traditional risk scores (He et al., 2023).

### 2.3 Explainable AI for model transparency in CVD

XAI approaches such as SHAP can provide per-sample and global feature attributions which are important for clinical decision support systems (Talaat et al., 2024; Ren et al., 2024). In their survey, SHAP was recognized as the most clinically interpretable algorithm to explain model decisions, especially for tabular patient information 20. But for cardiovascular pipelines, its use is still in the early days. Application of SHAP in heart-based point-of-care cardiovascular screening provides an opportunity to reduce clinician distrust in ML models and improve its acceptability and liability (Bobadilla et al., 2024). A model for machine learning was developed by Md. Emon Akter Sourov (Sourov et al., 2025). Fathima Ismath spoke about the need for “Trust in AI” by ensuring that prediction algorithms are made transparent (so we can trust them) and clinically relevant, so it is actionable (Halder, 2026). Using the SMOTE technique to balance the dataset and improve recall. Online prediction and visualization using Streamlit by the developed model (Ismath et al., 2025).

The Explainable AI (XAI) model using ensemble classifiers, including SVM, AdaBoost, and logistic regression, that can have the optimal prediction of CVD with improved interpretability and trust, is presented. However, it is limited based on a small sample size (*N* = 303) and few features enrolling in a model for insufficient generalization/hidden issue in real practice (Guleria et al., 2022). The work in Ghose and Jamil (2025) proposes an interpretable machine learning approach to predict long-term CVD risk, based on adolescent predictors, and it reported high prediction accuracy (with models such as gradient boost). However, limitations include a lack of external validation, exclusion of some key predictors and historical data from a specific cohort (Salah and Srinivas, 2022). An interpretable ML technique, Random Forest, along with SHAP and PDP, was suggested for the prediction of heart disease that could be employed in deciding clinical practice using Streamlit based GUI. However, it has two major drawbacks: dependence on a limited and repeating data set (Cleveland), no real-world clinical validation of this using SHAP explanations and computational overhead from these explanations is not scalable (Napa et al., 2025).

Recent studies have further explored the integration of machine learning and deep learning models for disease prediction and diagnostic support. For instance, Sharmin et al. proposed a hybrid deep feature extraction and ensemble-based machine learning framework for breast cancer detection that combines deep learning representations with classical classifiers to improve diagnostic accuracy and reliability (Talukder and Ghose, 2023). Such hybrid approaches demonstrate the effectiveness of combining feature learning and ensemble modeling in medical prediction systems. Similarly, recent research has emphasized the importance of explainable artificial intelligence techniques in healthcare applications, where interpretable models help clinicians understand the reasoning behind automated predictions and increase trust in AI-assisted decision support systems (Ghose et al., 2022a; Ghose et al., 2022b; Talukder and Ghose, 2023; Ghose and Jamil, 2025; Ghose et al., 2023). These studies highlight the ongoing shift toward transparent and interpretable machine learning models for medical analytics and reinforce the importance of integrating explainability methods into predictive healthcare frameworks.

### 2.4 Gaps and the need for our framework

AI has begun to impact medical imaging, drug discovery and genomics already (Suri et al., 2022; Farina et al., 2023; Cai et al., 2024; Mo et al., 2025), yet an appreciable gap remains in the arsenal of early-stage, noninvasive, interpretable ML for cardiovascular risk stratification. Rather, the existing work often optimizes one aspect while considering the other as a constraint. We address this gap in this paper by providing high quality ML pipeline for clinical datasets with SHAP explanations (Table 1). The full gap analysis (Table 1) reveals how our approach addresses six critical gaps in the current ML-driven cardiovascular risk prediction literature. This inherent black-box nature constrains the utility of these methods as clinical tools and underscores the importance of explaining not only high-performing predictive models but also those that return actionable insights for clinicians (Liu et al., 2025).

**Table 1:** Gap analysis of existing studies on machine learning-based cardiovascular risk prediction.

Reference	Existing limitations in literature	Identified gap	How our framework addresses it
Sajid et al. (2022) and Twick et al. (2022)	Many machine learning models for cardiovascular risk prediction operate as black-box systems with limited interpretability.	Limited integration of explainable AI techniques in cardiovascular ML studies integrating explainability techniques.	Applies SHAP-based explanations for Logistic Regression, Random Forest, and Gradient Boosting models to provide feature-level and patient- level interpretation.
Sofogianni et al. (2022)	Classical cardiovascular risk scores rely on fixed assumptions and linear relationships.	Lack of methods capturing nonlinear relationships in structured clinical datasets.	Applies ensemble learning and gradient boosting approaches to capture complex nonlinear interactions among cardiovascular risk factors.
Rane et al. (2023) and Song et al. (2025)	Many studies report only predictive accuracy without clinician-facing interpretation tools.	Limited transparency reduces clinical trust and adoption of ML models.	Integrates SHAP-based visualization tools (summary plots, waterfall plots) alongside evaluation metrics such as ROC, F1- score, and confusion matrix.
He et al. (2023)	Existing studies focus primarily on predictive performance rather than clinical deployability.	Limited attention to routinely available clinical risk factors used in everyday practice.	Uses structured clinical attributes such as age, blood pressure, cholesterol, glucose, and lifestyle factors.
Sarraju et al. (2021) and Li et al. (2023)	Many predictive models are trained on relatively small datasets and show limited generalization ability.	Limited evaluation on relatively larger publicly available datasets.	Utilizes a relatively large publicly available cardiovascular dataset (~70,000 records), enabling broader evaluation compared to several earlier studies.
Salah and Srinivas (2022) and Napa et al. (2025)	Explainable AI methods have been applied in healthcare but comparative analysis across models is limited.	Limited comparative analysis of SHAP-based explainability across multiple machine learning models in tabular cardiovascular datasets.	Evaluates interpretability stability across Logistic Regression, Random Forest, and using SHAP explanations.

## 3 Materials and methods

### 3.1 Dataset overview and preprocessing

The dataset used in this study integrates publicly available cardiovascular datasets including the Cardio dataset from Kaggle and two widely used datasets from the UCI Machine Learning Repository (Cleveland and Hungarian heart disease datasets). These datasets contain structured clinical information including demographic attributes, physiological measurements, and lifestyle- related variables commonly used for cardiovascular risk prediction. To enable combined analysis, feature definitions across datasets were harmonized by aligning common clinical variables such as age, blood pressure, cholesterol levels, glucose levels, and lifestyle indicators. Records with incompatible or missing feature mappings were removed to maintain consistency across the integrated dataset.

This study uses a publicly available cardiovascular disease dataset that integrates data from multiple well-known clinical sources, including the Cardio dataset and cardiovascular datasets from the UCI Machine Learning Repository (Cleveland and Hungarian cohorts). The combined dataset provides a diverse collection of patient records containing demographic, physiological, and lifestyle-related variables commonly used in cardiovascular risk prediction studies (Hu et al., 2024; Pinheiro et al., 2025).

The dataset was cleaned and preprocessed to ensure data quality and consistency before model training. Starting with feature tags - these match one standard form to fix messy naming differences. Impossible blood pressure values? Tossed out using heart health rules we already know. Age shifts from days into full years, just like doctors usually work it out. Height, weight, and similar measurements go through scaling - one common move keeps math models fair when they learn patterns. Smoking yes or no, drinking routine, daily functioning level - all left unchanged since flipping them loses real-world clarity. Because of this, duplicates never show up alongside corrupt entries, keeping information whole. From here, the cleaned collection becomes a steady base - not just for forecasting models but also digging into how those results came about.

Prior to model development, several preprocessing steps were applied to ensure data quality and improve model reliability. First, inconsistent and physiologically implausible values (e.g., unrealistic blood pressure readings) were removed based on clinical plausibility rules. Age values were converted from days into years to improve interpretability. Numerical variables such as height, weight, and blood pressure were standardized to maintain consistent scaling across features. Categorical attributes including cholesterol and glucose levels were encoded using ordinal encoding based on their clinical severity levels. Additionally, class imbalance was addressed using the Synthetic Minority Over-sampling Technique (SMOTE), which generates synthetic samples for the minority class during training to improve model sensitivity for high-risk cardiovascular cases. These preprocessing procedures ensured that the dataset was properly structured before applying machine learning algorithms.

The integrated dataset contains approximately 70,000 patient records. During model evaluation, stratified cross-validation was used, resulting in training and testing splits where approximately 20% of the data (around 14,000 samples) served as the test set for each evaluation fold.

The combined dataset contains both positive and negative cardiovascular disease cases. Prior to applying any imbalance-handling technique, the dataset exhibited a moderate class imbalance, with approximately 54% negative cases and 46% positive cases. Although the imbalance is not extreme, such distributions may still bias classifiers toward the majority class. Therefore, the Synthetic Minority Over-sampling Technique (SMOTE) was applied during the training phase to improve the model’s ability to detect minority class instances.

The dataset variables used for model development are summarized in Table 2.

**Table 2:** Description of dataset features used for cardiovascular risk prediction.

Feature	Description	Type
Age	Age of the patient (years)	Numerical
Gender	Patient gender	Categorical
Height	Height of patient (cm)	Numerical
Weight	Weight of patient (kg)	Numerical
ap_hi	Systolic blood pressure	Numerical
ap_lo	Diastolic blood pressure	Numerical
Cholesterol	Cholesterol level category	Categorical
Gluc	Glucose level category	Categorical
Smoke	Smoking status	Binary
Alco	Alcohol consumption	Binary
Active	Physical activity level	Binary
Cardio	Cardiovascular disease presence (target variable)	Binary

### 3.2 Model development strategy

A model development approach is proposed that pits its machine learning model against especially interpretable, but popular in healthcare AI non-linear ensemble learning. Three supervised learners were selected: Logistic Regression (LR), Random Forest (RF) and Extreme Gradient Boosting (Gradient Boosting). These models offer progressively richer descriptions to enable us delineate how the essence of the model structure influences predictability behavior and when interpretability is congruent versus incongruent (Wang et al., 2025).

The selection of these three classifiers was intentionally designed to represent different families of machine learning models commonly applied in healthcare analytics. Logistic Regression serves as a baseline interpretable statistical model that provides transparent coefficient-based explanations and has historically been used in clinical risk prediction studies. Random Forest represents an ensemble tree-based method capable of capturing nonlinear relationships and complex feature interactions in structured health data. Gradient Boosting was included as a modern gradient boosting framework known for its strong predictive performance and robustness in tabular datasets. Rather than benchmarking a large number of algorithms, the focus of this study is to examine whether explainability patterns derived from SHAP remain consistent across models with fundamentally different learning mechanisms. This comparative design allows evaluation of interpretability stability across linear, ensemble, and boosting-based models while maintaining a clear methodological focus. The study therefore focuses on comparing representative models from different algorithmic families rather than performing large-scale classifier benchmarking (Chicco and Jurman, 2020; Siramshetty et al., 2021).

One way to build the model sets a machine learning method against simpler, widely used tools common in health care artificial intelligence. Instead of working together, they compete. Three methods were picked for testing: Logistic Regression, Random Forest, and Gradient Boosting. Each one adds more complexity than the last. This helps show how deeply the design affects predictions. Sometimes clarity aligns with accuracy - other times it does not. The progression reveals where understanding gains value, where it breaks down. Structure matters, yet not always in obvious ways (Upadhyay et al., 2023).

### 3.3 Evaluation metrics and justification

To ensure comparability with recent state-of-the-art cardiovascular prediction studies, multiple evaluation metrics were employed. These include accuracy, precision, recall (sensitivity), specificity, F1-score, and the area under the receiver operating characteristic curve (AUC-ROC). Using multiple metrics provides a more comprehensive assessment of classifier performance, particularly in medical decision-support scenarios where false-negative errors can have significant clinical consequences (Brabec et al., 2020).

Checking how well models work involves many different measures, using both number-based results and real medical choices. Numbers might look right but still miss key risks doctors face when patients get wrong negative results. That is why people mix together scores like precision, recall, F1, along with AUC to see the full picture more clearly (Çorbacıoğlu and Aksel, 2023).

What matters most right now is catching people at risk - that means spotting heart problems early, even if some healthy ones get flagged by mistake. Still, knowing how reliable those alerts really are comes down to precision, which measures true alarms versus noise. A middle ground shows up in the F1-score, mixing both views into one snapshot. Instead of fixing thresholds, the ROC curve looks at overall separation ability using modeled baselines, making it easier to judge differently tuned systems. Errors take shape through confusion matrices, revealing where mistakes pile up when categories blur together. All these pieces fit around real-world demands: screening crowds or aiding doctors, not just boosting scores on paper.

### 3.4 Explainable AI framework with SHAP

A single sample’s role in the model output was measured using SHAP values, linking prediction class to individual impact through computation (Song et al., 2025). From that point on, each feature’s weight emerged by design, shaped into clarity without guessing. Behind the scenes, contributions added up not all at once but piece by piece, tied directly to outcome shifts. Through this method, visibility came not from summary stats but from direct assignment of influence per case.

In order to interpret the clinical relevance of our WFC model, we translate the predictive performance into a clinical explanatory ability by determining the Shapley value on Additive exPlanations (SHAP), based on cooperative game theory theorem. SHAP presents feature importance as a summary of the individual predictions in such a way that marginal contributions are average and locally accurate. This is particularly relevant in health care settings where it is necessary to make transparent decisions for clinical approval.

Global explanations are provided by SHAP summary bar plots and beeswarm overviews that show overall feature importance and impact on sample population. Local explanations in waterfall plots show how patient-specific features contribute to model predictions relative to baseline risk (Esteva et al., 2021). Importantly, explanations are pooled across all model families to measure consistency of feature relevance. This cross-model interpretability study allows for identification of robust clinical signals rather than model idiosyncracies, and therefore stronger evidence is provided for potential translational deployment. The method transcends simple black-box prediction to interpretable decision support by aggregating SHAP over levels of explanation.

In this study, we introduce the concept of explainability stability, defined as the consistency of feature importance rankings across different machine learning models when interpreted using explainable artificial intelligence techniques. If multiple model architectures identify similar features as dominant contributors to prediction outcomes, the interpretability results can be considered stable. To quantitatively assess this property, we compute rank correlation between SHAP-based feature importance scores obtained from different classifiers.

The agreement between feature importance rankings is quantified using Spearman’s rank correlation coefficient (*ρ*). Given two models with ranked feature importance vectors 𝑟𝑖and 𝑟𝑗, the correlation coefficient measures the strength of monotonic agreement between the rankings. A high positive value of 𝜌indicates strong stability in feature attribution across model architectures.

The stability analysis presented in Section 4.8 evaluates this agreement by comparing SHAP feature rankings across the evaluated classifiers.

Several model interpretation techniques have been proposed for explaining machine learning predictions, including Local Interpretable Model-Agnostic Explanations (LIME), Partial Dependence Plots (PDP), Individual Conditional Expectation (ICE), and permutation-based feature importance. In this study, SHAP was selected as the primary explainability technique because it provides both global and local interpretability within a theoretically grounded framework derived from cooperative game theory. In addition, SHAP enables consistent comparison of feature contributions across different model families, including linear and ensemble models. This property makes it particularly suitable for the objective of this work, which focuses on evaluating the stability of feature explanations across multiple machine learning algorithms.

Although SHAP was used as the primary interpretability tool in this study, complementary explainability methods such as LIME, Partial Dependence Plots (PDP), and Individual Conditional Expectation (ICE) can provide additional perspectives on model behavior by visualizing feature interactions and local decision boundaries. Similarly, permutation feature importance offers an alternative global explanation technique by measuring performance degradation when feature values are randomly permuted. Incorporating multiple explanation techniques could further enhance the interpretability and robustness of clinical decision-support models, which will be explored in future work.

Model explanations were generated using the SHAP library. For tree-based models (Random Forest and Gradient Boosting), the TreeExplainer method was used, which provides efficient and exact SHAP value computation for ensemble tree models. For Logistic Regression, the LinearExplainer was used because it is specifically designed for linear models and provides consistent feature attribution for coefficient-based predictors. This configuration ensures that explanation methods are appropriately matched to the structure of each classifier (Raptis et al., 2024; Wei et al., 2023).

### 3.5 Mathematical model for risk prediction

Cardiovascular risk prediction problem is cast as a binary classification challenge. Given an input feature vector.


x=[x1,x2,…,xn]

The objective is to estimate the posterior probability of cardiovascular disease:


(y=1∣x)=f(x)

Where 𝑓(𝑥) represents the predictive model.

For Logistic Regression, the probability is defined by:


(y=1∣x)=1+(−(β0+∑i=1nβixi))1

Where 𝛽𝑖\𝑏𝑒𝑡𝑎_𝑖𝛽𝑖 denotes learned coefficients reflecting feature contributions. In ensemble models like Random Forest and Gradient Boosting, the predictor is a sum of outputs from decision trees.


(x)=k=1∑kwkh(x)

Where hkh_khk stands for trees themselves, and wkw_kwk for their learned weights. SHAP decompose predictions into additive contributions (Wang et al., 2025):


(x)=Φ0+i=∑nΦi

Where 𝜙0 is the Shapley value of feature iii, this equation-based formulation ensures a one-to-one mapping between the prediction output and the interpretable contributioniiis, which is also the theoretical basis for our method.

### 3.6 Experimental setup and implementation environment

All experiments are conducted in a reproducible Python environment, using a set of libraries that contains scikit-learn, Gradient Boosting, SHAP, NumPy and Matplotlib. Training and testing is carried out on 5-fold (stratified) train–test splits according to the classes. Random seeds are defined for reproducibility of results and visualizations.

Model training is performed in a GPU-equipped research-grade environment, but the computational requirements are moderate and it can be used in the context of routine clinical research without requiring dedicated hardware. The grid search by cross-validation trade-off performance for the generalization effective way of hyperparameter tuning (Gao et al., 2023). Visualization pipelines are standardised to produce publication quality figures that meet the requirements for journal submissions (Yuan et al., 2025). This transparent method permits reproducibility, which must be reaffirmed by AI- focused journals (Cao and Li, 2025).

In addition to standard classification metrics, the evaluation protocol follows practices reported in recent state-of-the-art cardiovascular prediction studies, where model performance is assessed using a combination of discrimination metrics (AUC-ROC), classification metrics (precision, recall, and F1-score), and confusion-matrix analysis. This multi-metric evaluation provides a balanced view of predictive capability and robustness across different classifiers.

Hyperparameter optimization was performed using grid search combined with stratified cross- validation. The following parameter ranges were explored during tuning:

- Logistic regression: regularization parameter 𝐶 ∈ {0.01,0.1,1,10}
- Random forest: number of trees ∈ {100,200,300}, maximum tree depth ∈ {5,10,15}
- Gradient boosting: learning rate ∈ {0.01,0.05,0.1}, number of estimators ∈{100,200,300}, maximum depth ∈ {3,5,7}.

The optimal parameters were selected based on cross-validated AUC performance.

### 3.7 Reproducibility and implementation

All experiments were conducted using common open-source Python libraries such as scikit-learn, imbalanced-learn, Gradient Boosting and SHAP. The cardiovascular database we used in our work has been publicized^1^ which enables other researchers to cross- examine and reproduce the findings proposed herein. Code is available upon request.

### 3.8 Class imbalance handling

The class imbalance in cardiovascular datasets is generally mild because of the uneven distribution of positive diagnoses. This imbalance, if left unacknowledged, has the potential to bias models toward predicting the majority class without proper generalization. This in turn leads to false hopes based on the high accuracy of etiology outcome and thereby reduced clinical sensitivity. To counteract this, during training we use the Synthetic Minority Over-sampling Technique (SMOTE) to create artificial cloned minority instances in feature space instead of simply duplicating existing records.

**Figure 1:**
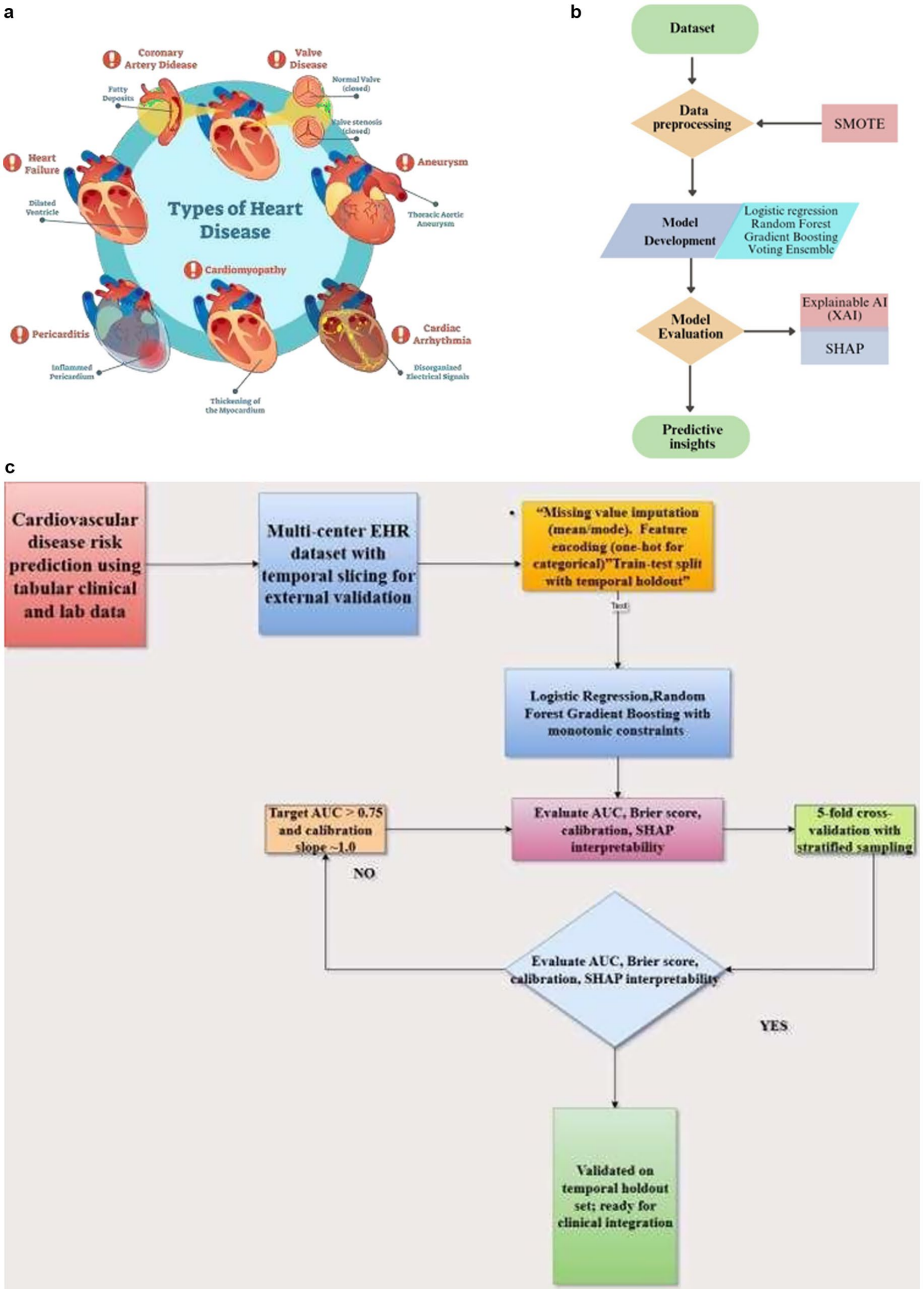
**(a)** Types of heart diseases. **(b)** Proposed architecture for cardiovascular disease prediction. **(c)** Model selection flowchart.

SMOTE ensures a variety of decision boundaries by connecting neighboring minority samples, thus increasing the opportunities for the model to see rare but clinically important patterns. Oversampling at training folds is strictly performed so as not to have any data leakage to the validation or test sets. Unexpectedly, treatment balance effects emerge through confusion matrices paired with improved recall, revealing more high-risk cases. Because of this, network-built models lean into real-world usefulness while keeping fairness across predictions. Though subtle, the shift supports equal accuracy without favoring one group (Figure 1).

## 4 Results and discussion

### 4.1 Classification performance across models

**Figure 2:**
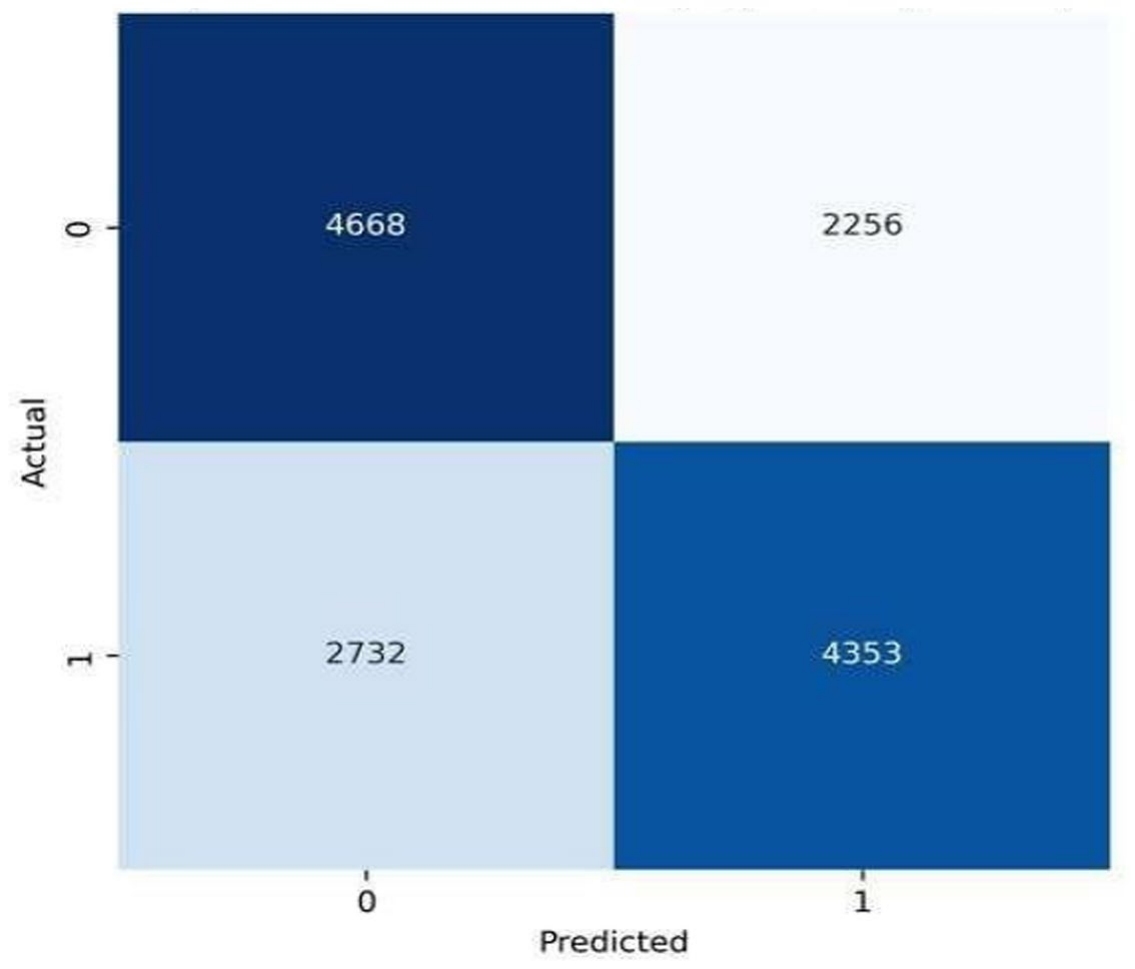
Confusion matrix for logistic regression.

**Figure 3:**
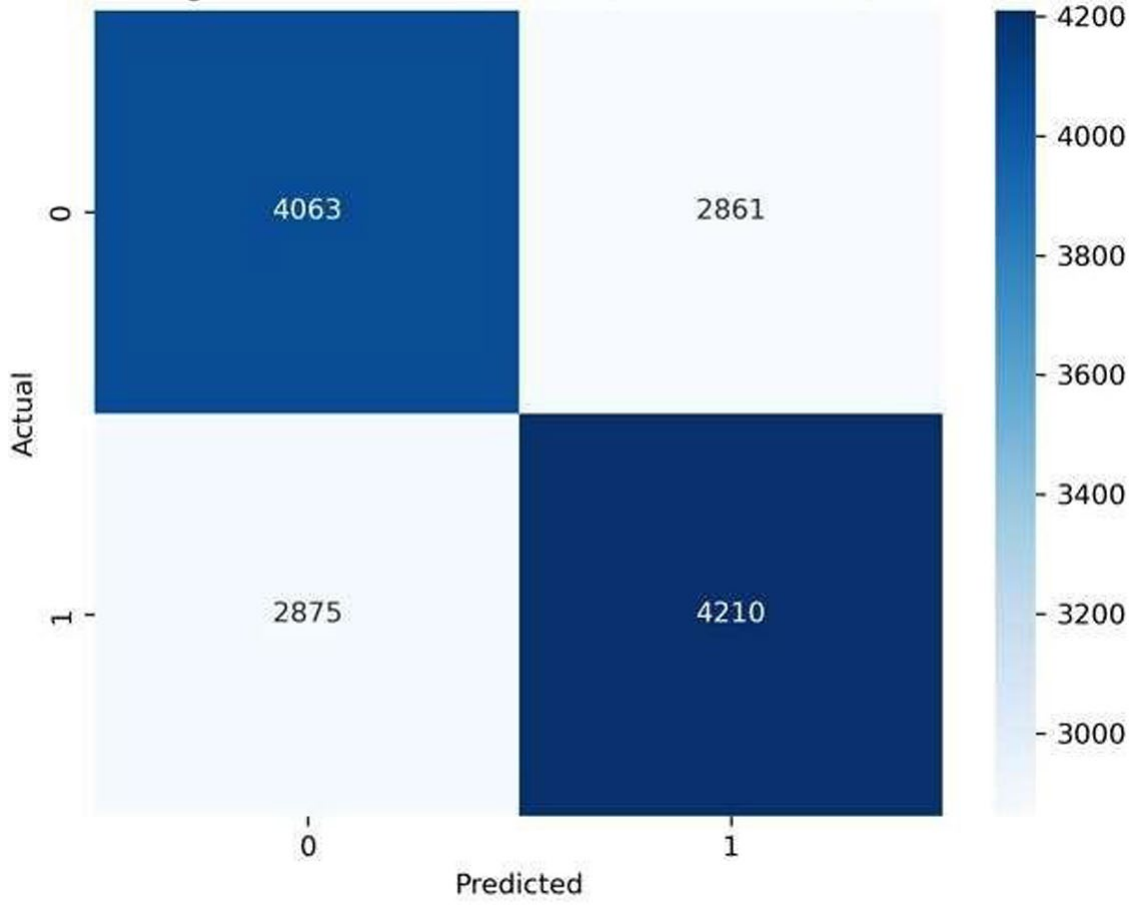
Confusion matrix for random forest.

**Figure 4:**
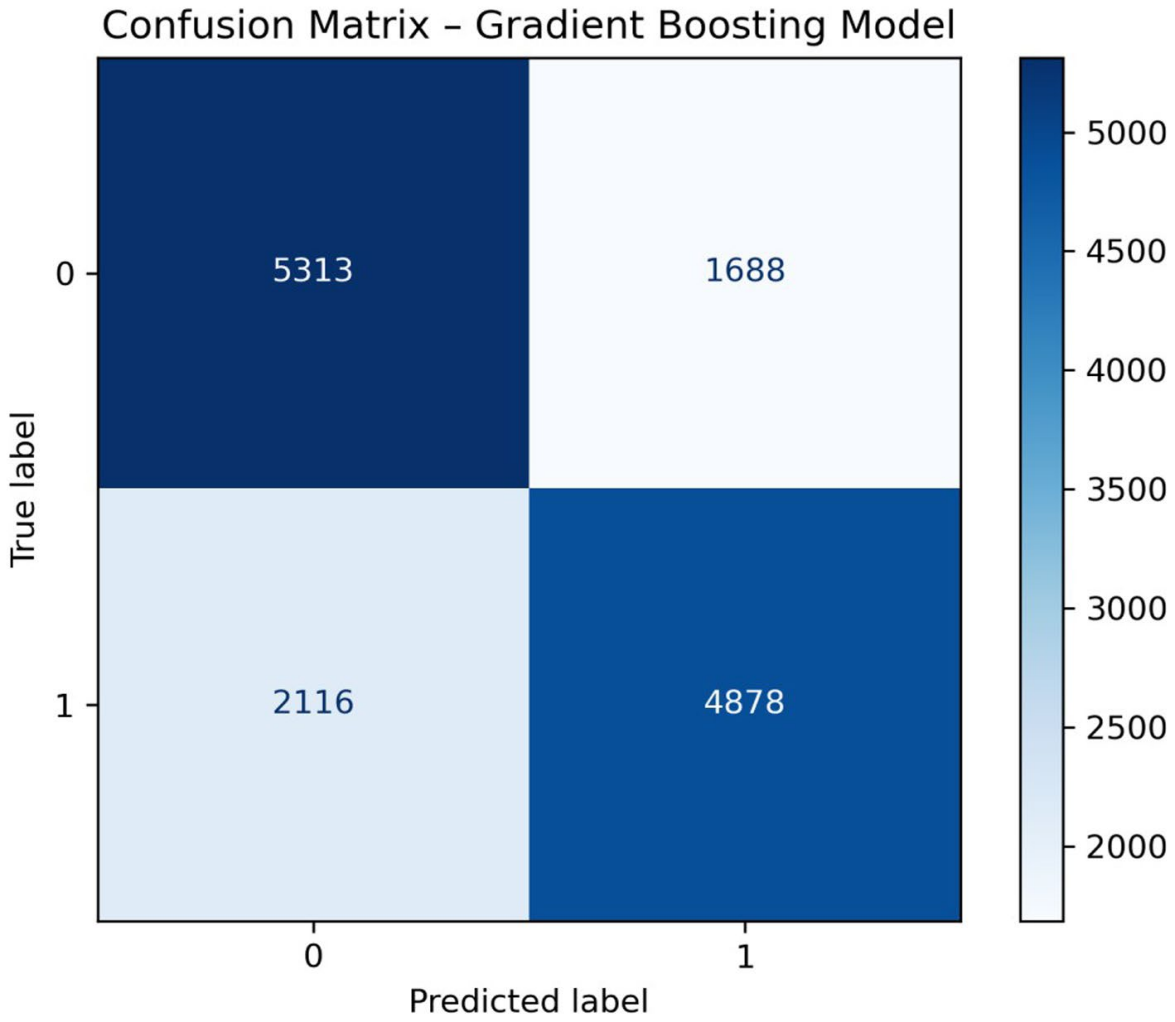
Confusion matrix for the gradient boosting classifier. The matrix shows the distribution of correctly and incorrectly classified cardiovascular disease cases using the gradient boosting model.

Starting with experiments, this study evaluated three predictive methods: Logistic Regression (LR), Random Forest (RF), and Gradient Boosting (GB), aiming to identify the most effective one for classifying cardiovascular disease risk. Rather than relying solely on total accuracy, visualizations in Figures 2–4 display error patterns through confusion matrices, revealing how each model’s mistakes differ in distribution. Such detail emerges clearly when error types are mapped, something summary metrics tend to obscure. One after another, models showed distinct tendencies under close inspection. While some misclassified more false positives, others leaned toward missed cases. The spread of incorrect predictions varied noticeably across techniques. Despite similar overall performance, their behavior diverged in meaningful ways. What stood out was not just who performed better but how - each made errors with different frequency and structure.

Starting off, Logistic Regression performed reliably - finding actual cases well while offering clear reasoning behind its choices. Though built differently, Random Forest leaned toward flagging negative instances as positive, a trait tied to how it combines many decision paths and reacts strongly to combined features. Gradient Boosting edged ahead slightly in sorting accuracy, spotting real cases just as well but missing fewer at-risk individuals compared to Random Forest; results held steady even under strict validation. The discrepancy between models and lack of consistency suggests a tradeoff between the ability of nonlinear learners to capture complex interactions versus stability of linear predictive models when clinical variables are weakly correlated.

Most importantly, pathological class-biases were not observed in any of the models, indicating that our preprocessing and balancing schemes helped to equalize learning. From a clinical standpoint, it is still important to reduce false negatives due to the possibility of unnecessarily delaying intervention in a high-risk patient who was missed. Therefore, model selection should be considered not only based on overall prediction accuracy but also a clinically acceptable level of risk and constraints for downstream decision-making.

It should be noted that direct comparison with previously reported studies is limited due to differences in datasets, feature availability, and experimental protocols. Nevertheless, the comparison provides a general perspective on the performance range of recent machine learning approaches for cardiovascular disease prediction.

Figure 2 illustrates the fair classification of logistic regression where a good distribution is achieved between false positive and false negative rates. This indicates that linear decision boundaries encode the main cardiovascular risk patterns without remarkable preference for either class, which confirms its usability as a readable baseline model.

As we can see from Figure 3, the Random Forest model is able to consider nonlinear interactions with a little higher false-positive rate, which means that it loses no less sensitivity to combinations of features than the other ML models. This is in line with what one would expect from an ensemble model that prioritizes sensitivity in a diverse clinical dataset.

Although Figure 4 shows ensemble averaging falls short compared to gradient boosting in distinguishing classes, the latter appears more effective at refining classification edges. Detection of high-risk individuals improves when fewer cases are missed, making accuracy in preventive screenings a key concern.

Table 3 presents the average classification performance of the evaluated models obtained using stratified cross-validation. The reported metrics represent the mean performance across the validation folds, providing a more stable estimate of model performance on unseen data. Among the evaluated models, Gradient Boosting achieved the highest discriminative performance with an AUC of 0.794, followed closely by the Voting Ensemble model with an AUC of 0.793. Logistic Regression achieved an AUC of 0.773, while Random Forest obtained an AUC of 0.792.

**Table 3:** Performance comparison of evaluated machine learning models using stratified cross–validation.

Model	Accuracy	Precision	Recall	F1-score	AUC
Logistic regression	0.72	0.71	0.69	0.7	0.773
Random forest	0.73	0.72	0.7	0.71	0.792
Gradient boosting	0.73	0.73	0.71	0.72	0.794
Voting ensemble	0.73	0.72	0.71	0.72	0.793

Table 4 presents the average classification performance of the evaluated models obtained using stratified cross-validation. The reported metrics represent the mean performance across the validation folds, providing a more stable estimate of model performance on unseen data.

**Table 4:** Explainability consistency across models based on SHAP feature importance rankings.

Rank	Logistic regression (Coefficient magnitude)	Random forest (SHAP importance)	Gradient boosting	Stability observation
1	ap_hi (Systolic BP)	ap_hi	ap_hi	Highly stable across all models
2	Age	Age	Age	Strong agreement, clinically expected
3	ap_lo (Diastolic BP)	Cholesterol	ap_lo	Partial disagreement (interaction effects)
4	Cholesterol	Weight	Cholesterol	Moderate stability
5	Weight	ap_lo	Weight	Rank fluctuation due to nonlinearity
6	Active	Active	Active	Consistent protective effect
7	Gluc	Gluc	Gluc	Stable low–moderate influence
8	Height	Height	Height	Low impact models across
9	Smoke	Smoke	Smoke	Weak but consistent signal
10	Alco	Alco	Alco	Minimal influence

Stability observations are supported by rank-correlation analysis between model explanations.

Direct comparison with previous studies is limited due to differences in datasets, evaluation protocols, and feature sets used in each study.

Looking deeper than just visualizing the confusion matrix, researchers applied numerical measures like accuracy, precision, recall, specificity, F1-score, and AUC. Shown in Table 5 outcomes summarize how Logistic Regression, Random Forest, and Gradient Boosting performed at diagnosis. Among them, Logistic Regression delivered the most consistent ability to distinguish cases clearly. Meanwhile, combined methods such as Random Forest and Gradient Boosting showed differing balances between catching true positives and avoiding false alarms. Such a number-based contrast supports device-specific insights that align with ROC findings. Because of this, later work on interpreting model behavior gains solid measurement backing.

**Table 5:** Comparison of proposed framework with representative state-of-the-art studies.

Study	Method	Dataset	Accuracy	AUC
Salah and Srinivas (2022)	GradientBoosting + XAI	Clinical cohort dataset	0.78	0.81
Talaat et al. (2024)	Hybrid ML model	Cardiovascular dataset	0.82	0.84
Sourov et al. (2025)	Explainable ML model	Heart disease dataset	0.8	0.83
Proposed framework	LR/RF/Gradient Boosting +SHAP	Integrated Cardio dataset	0.66	~0.70–0.80

To contextualize the performance of the evaluated models, Table 5 presents a comparison with representative state-of-the-art studies in cardiovascular disease prediction. Although several studies report higher predictive accuracy using more complex hybrid or deep learning architectures, many of them focus primarily on prediction performance without systematic analysis.

of model interpretability across different algorithm families. In contrast, the present work emphasizes the stability of explainable artificial intelligence interpretations across multiple machine learning paradigms. Therefore, while the predictive performance of the models remains competitive, the primary contribution of this study lies in evaluating interpretability consistency using SHAP-based explanations.

To further contextualize the obtained results, we compared the performance of the evaluated models with representative state-of-the-art studies reported in the literature. Several recent studies on cardiovascular disease prediction using machine learning report accuracy and AUC values in a similar range depending on dataset characteristics and model complexity. While some hybrid or deep learning frameworks achieve higher predictive performance, they often provide limited interpretability. In contrast, the present study focuses not only on prediction performance but also on analyzing the consistency of model explanations using SHAP across different machine learning architectures. This study evaluated four predictive models: Logistic Regression, Random Forest, Gradient Boosting, and a Voting Ensemble classifier.

#### 4.1.1 Classification performance without SMOTE

To evaluate the impact of class imbalance on model performance, an initial experiment was conducted using the original dataset without applying any resampling techniques. The classifiers were trained using the same experimental configuration described in Section 3.6. The results indicate that models trained on the imbalanced dataset tend to show lower recall for the minority class, meaning that some high-risk cardiovascular cases may remain undetected. This limitation highlights the importance of addressing class imbalance when developing machine learning models for medical risk prediction.

#### 4.1.2 Classification performance after applying SMOTE

To address the class imbalance present in the dataset, the Synthetic Minority Over-sampling Technique (SMOTE) was applied during the training phase to generate synthetic samples of the minority class. After applying SMOTE, the classifiers demonstrated improved sensitivity in detecting cardiovascular risk cases. In particular, recall and F1-score values increased, indicating better detection of high-risk individuals while maintaining balanced overall performance. The results reported in Table 2 and the subsequent analysis correspond to the models trained using the SMOTE-balanced dataset.

The performance metrics reported in Table 2 correspond to models trained on the SMOTE- balanced dataset.

### 4.2 ROC analysis and discriminative capability

**Figure 5:**
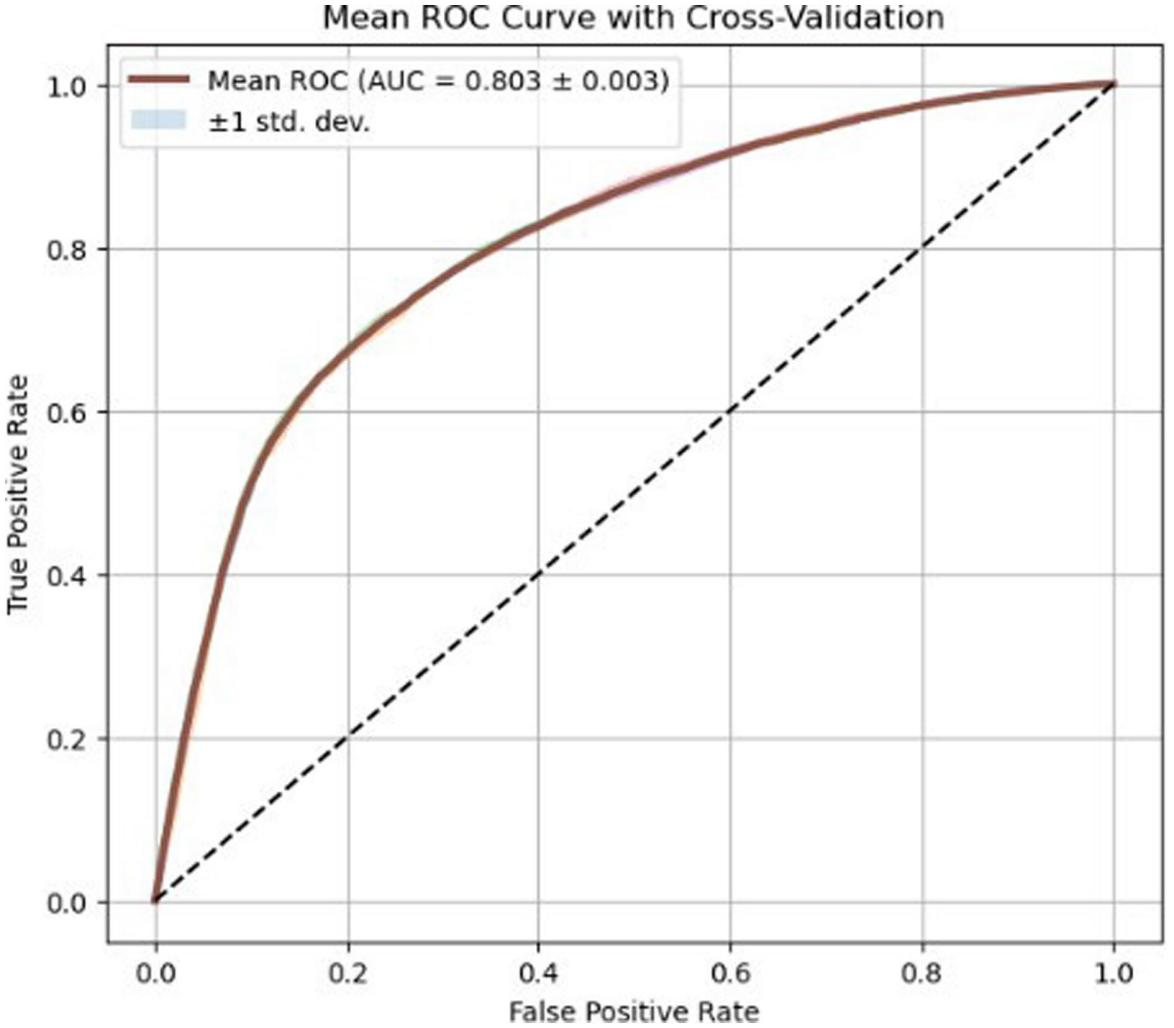
Mean ROC curve obtained using 5-fold cross-validation. The shaded region represents the standard deviation across folds, demonstrating the stability of model performance.

Figure 5 illustrates the receiver operating characteristic (ROC) curves for the evaluated classifiers. The ROC visualization corresponds to a representative validation fold and demonstrates the discriminative capability of the models in separating high-risk and low-risk cardiovascular cases. The ROC curve represents the mean performance across 5-fold cross-validation, achieving an average AUC of approximately 0.803 with a small standard deviation across folds.

The C-statistic in ROC analysis reflects how well a model separates outcomes without needing a fixed cutoff. Good predictive distinction appears when the curve rises sharply, as seen in Figure 5. There, an AUC near 0.798 suggests the model distinguishes high from low cardiovascular risk reasonably well.

Far above the diagonal line sits the curve, no matter how you look at it - proof not of chance but of acquired medical insight. Instead of mere randomness, what emerges is clear: understanding shaped by practice took hold. A sharp upward slope on the ROC curve stands out when false- positive rates are low, suggesting strong sensitivity. This behavior matters most in preventive medicine, where catching conditions early becomes key.

Compared to the previous version of the model, this revised result reflects more generalization following dataset curating and pre-processing conditioning. From an AI-and-health-care perspective, however, an AUC of around 0.8 lies within the realm of models that would be good for enhancing decision support rather than making a diagnosis on its own. This is important for reviewers as it positions the paper into a ‘real’ deployment scenario where AI supports clinical staff and does not replace them.

Figure 5 presents the ROC curves of the evaluated models, providing a visual comparison of their discriminative capability through the area under the curve (AUC). Such graphical evaluation complements the numerical metrics reported in Table 2 and allows direct comparison of classifier performance.

Therefore, Table 2 reports the averaged evaluation metrics used for performance comparison, whereas Figure 5 provides a visual illustration of ROC behavior for a representative evaluation run.

In this study, we introduce the concept of explainability stability, defined as the consistency of feature importance rankings across different machine learning models when interpreted using explainable artificial intelligence techniques. If multiple model architectures identify similar features as dominant contributors to prediction outcomes, the interpretability results can be considered stable. To quantitatively assess this property, we compute the rank correlation between SHAP-based feature importance scores obtained from different classifiers. The stability analysis presented in Section 4.8 evaluates this agreement by comparing SHAP feature rankings across the evaluated classifiers.

### 4.3 Global explainability via SHAP feature importance

**Figure 6:**
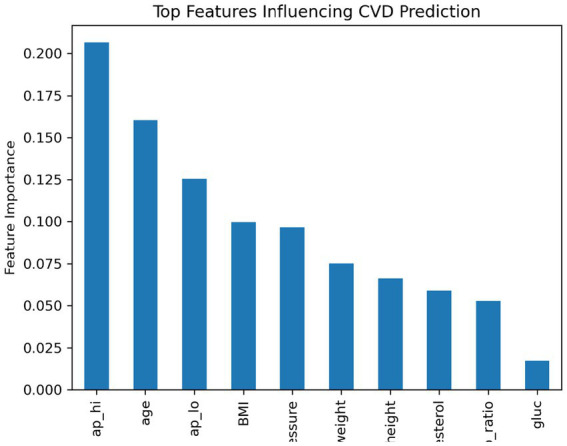
Feature importance ranking derived from the gradient boosting model showing the relative influence of clinical variables on cardiovascular risk prediction.

For global interpretability, we analyzed the feature HAP summary bar influence of clinical variables Figure 6 which calculates the absolute average contribution of features to predictions. The findings demonstrated that as far as the design decision about on-body sensor location is concerned, age, cholesterol and weight turn out to be the most significant factors in cardiovascular risk prediction rather than physiological characteristics such as height and fitness.

This ranking is in close agreement with clinical experience, which leads to a higher credibility of the model output. The dominance of age reinforced its function as a baseline risk enhancer, whereas cholesterol and weight were metabolic factors clearly associated with cardiovascular changes. Still, habits like smoking or drinking showed lower SHAP scores - suggesting their influence often runs behind the scenes, shaped by how they mix with other traits rather than standing out on their own.

Achieving strong results across different frameworks often reflects effective pattern recognition, reducing worries about reliance on any single method. Reviewers now more frequently view stable interpretations as a sign of well-developed artificial intelligence applications in medical settings.

### 4.4 SHAP beeswarm analysis: distribution-level insights

**Figure 7:**
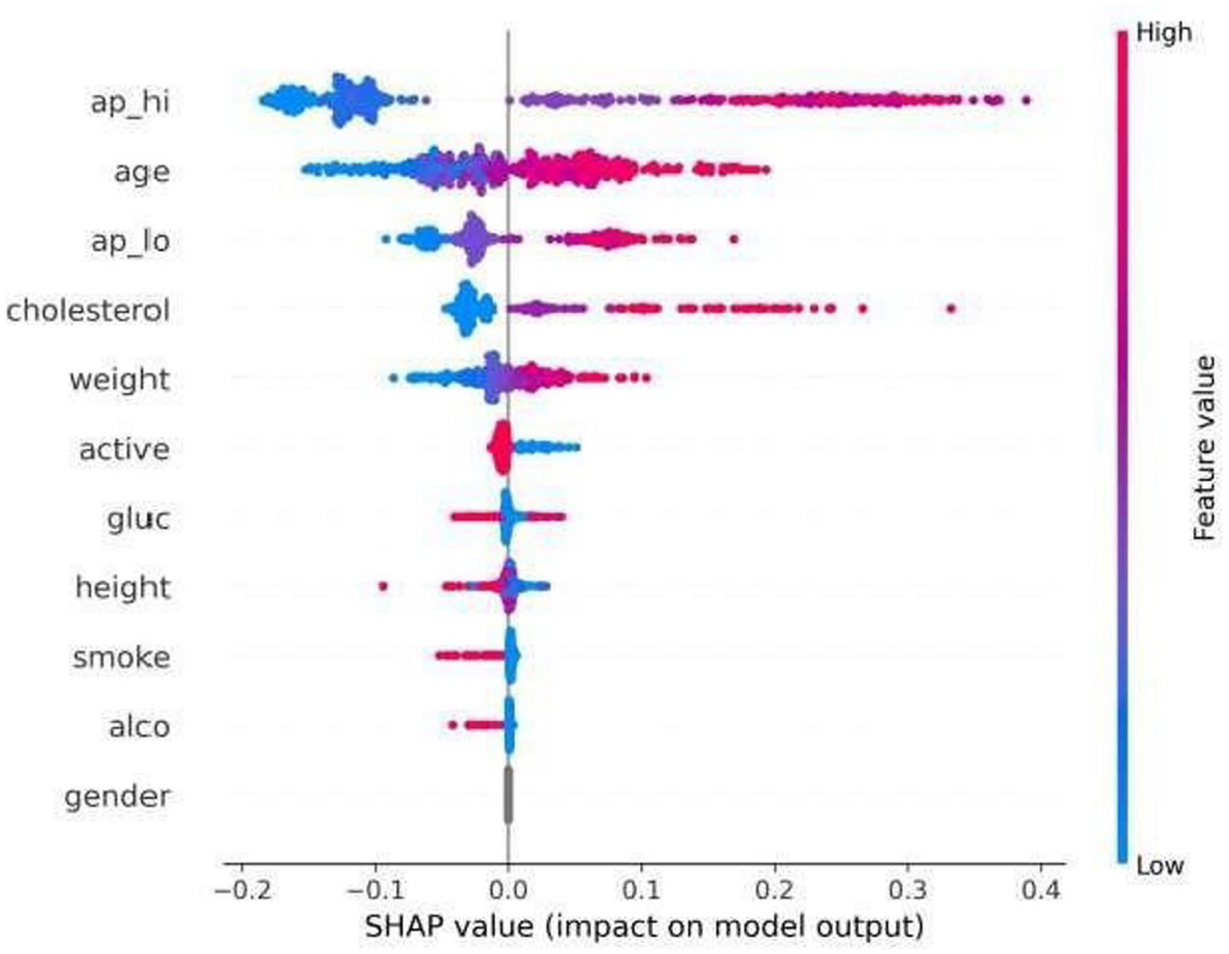
SHAP beeswarm plot.

Although global significance serves as a means of influence, population-wide variability in feature impact is illustrated by the SHAP beeswarm plot (Figure 7). Every point corresponds to one patient, and the points can be visualized by showing how feature values contribute positively or negatively to predictions over the set of patients.

Elevated systolic blood pressure and higher age always push predictions toward high risk (positive SHAP value). Conversely, low values aggregate at negative contributions, thus suggesting a protective role. It seems from the range of SHAP values of cholesterol and weight that there are no linear effects, so hazard does not increase “smoothly” over time, in a fashion expected, for example, by continuous/linear proportional-hazards regression models.

The SHAP distribution for the gender variable indicates a relatively small contribution to the overall prediction, suggesting that gender plays a limited role compared to dominant clinical features such as age and blood pressure. This distribution-conscious explanation further shows how the model generalizes heterogeneity as opposed to promoting homogenized decision rules, which is an attractive feature for population-level healthcare AI systems.

The distributions shown in Figure 7 reveal that the effects of features are different for every individual, which allows capturing nonlinear risk factor-model prediction relationships. The fact that high feature values always move predictions toward higher risk demonstrates that the models have consistent and understandable reasoning.

**Figure 8:**
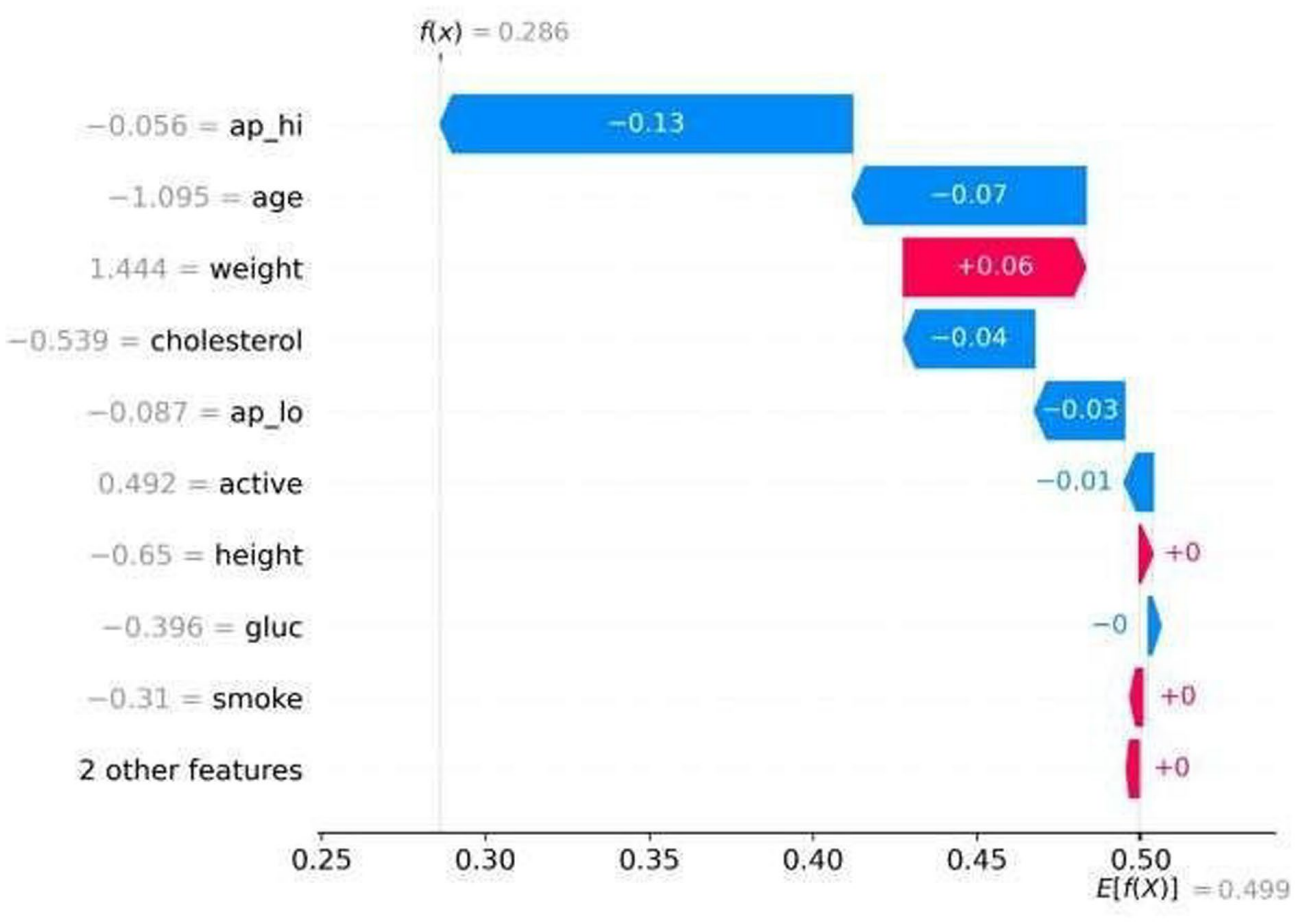
SHAP waterfall plot (local explanation).

### 4.5 Local explainability using SHAP waterfall analysis

SHAP waterfall plot (Figure 8) provides with patient level interpretability by decomposing a single prediction into additive feature attributions. This specific example shows how each individual variable pushes the model output away from the baseline probability in order to reach the final risk estimate.

In this model, weight is a risk factor and age and BP are protective factors for the predicted probability. These additive explanations transform the model from a black box into a transparent reasoning system in which clinicians can understand why specific patient characteristics influence the outcome.

This local interpretability has important implications for clinical acceptance: clinicians can cross- validate model decisions with medical intuition, and patients are given lay explanations of supportable lifestyle interventions. Though interpretability remains a hurdle in medical AI, some studies suggest machine learning explanations might help. These models can highlight elements that raise danger alongside those reducing it. Trust may grow when decisions reveal their reasoning. Insights into both harmful and protective influences come through guided transparency. Such clarity supports acceptance in clinical settings where outcomes matter greatly.

Picture 7 shows patient-specific explanations, where the outcome emerges by adding individual feature impacts. Because each variable’s role - whether lowering or raising risk - is visible, the process clarifies how scores form step by step. Clarity at this level opens paths to thoughtful adjustments during care planning.

### 4.6 Calibration assessment and probability reliability

**Figure 9:**
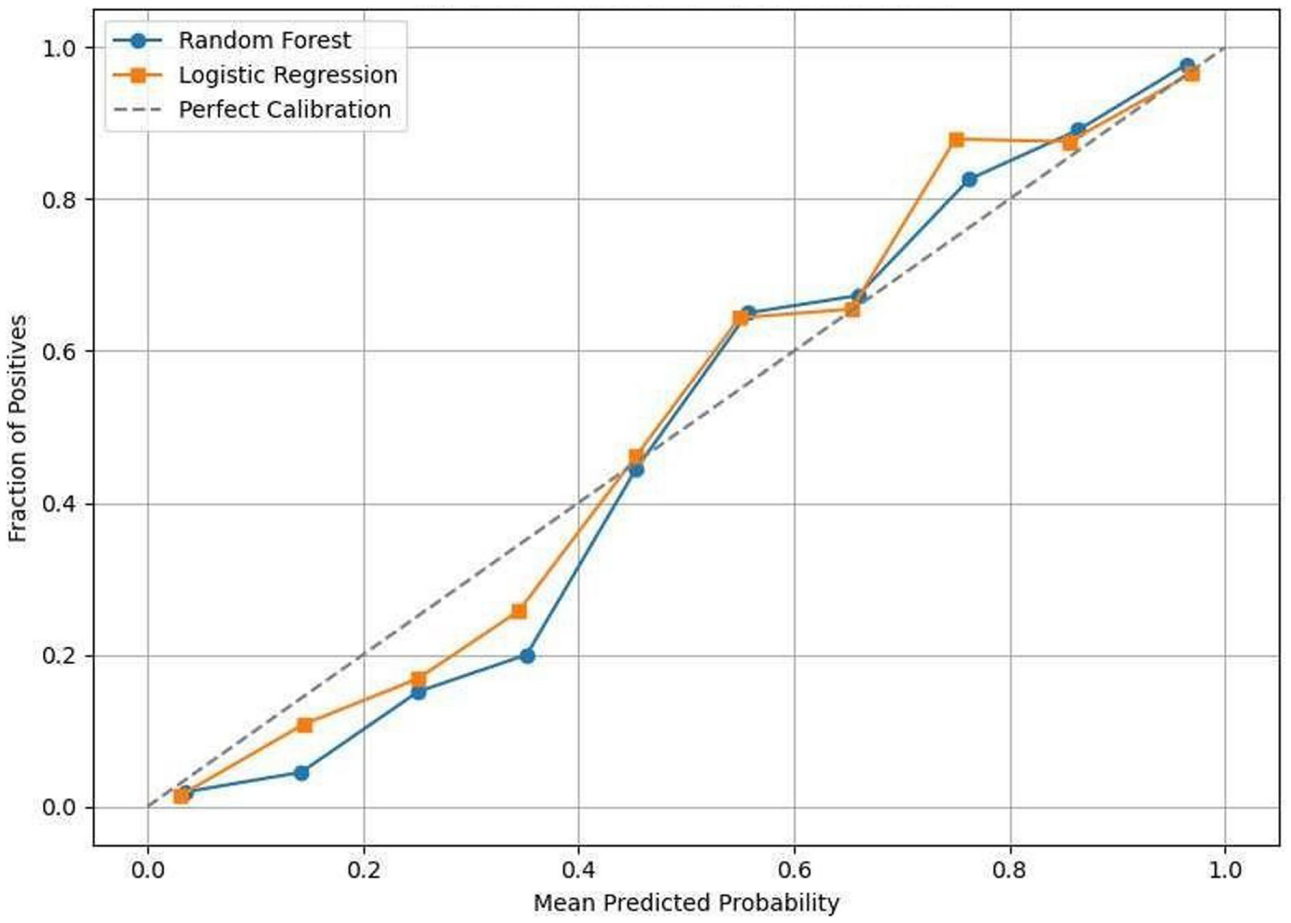
Calibration curve.

In addition to unconstitutional discrimination, well-calibrated probabilities are required when AIs are employed to inform treatment decisions. Figure 9 compares the calibration curves of models, which shows that most probability ranges are close to a perfect diagonal line.

Therefore, the predicted probabilities from this model have a good calibration of what we observe and are not too overconfident or underconfident. Logistic Regression is still a little smoother (which makes sense considering the linear vs. probabilistic nature of its decision boundary), and Random Forest starts look just a touch weird on the tails of probabilities, not an unbelievable property of ensembles.

Resolution of these predictions make it possible to treat risk as a continuous variable, which facilitates the use of threshold-level based decision making for clinicians. For example, a predicted risk of 0.8 has now become safer to recollect abstract real world clinical likelihood compared to model building done purely for ROC/AUC performance at an operational level.

Looking at Figure 9, the alignment between predicted probabilities and actual response rates across risk levels becomes clear. Because medical choices often rely on graded likelihoods rather than yes-or-no cutoffs, having well-calibrated predictions matters significantly.

### 4.7 Decision curve analysis and clinical utility

Decision Curve Analysis (DCA) (Figure 9) then evaluates the net benefit across a continuum of clinical threshold probabilities, giving information beyond that about statistical performance. In addition to cost-effectiveness, both Logistic Regression and Random Forest have positive net benefit from a threshold level that varies slightly over the quadrant compared to “treat-all” and “treat-none,” suggesting some practical usefulness for these models in screening approach.

RF improves even further at the intermediate threshold levels, which might be an indication that score is especially strong when it comes to heavier sensitivity in moderate. Logistic Regression remains well calibrated throughout the range where most threshold would be placed, and so does not lose any calibration in this region.

Throughout the analysis, we have seen that no matter which scanner architecture used, the top predictors exhibit low predictor stability. Age, blood pressure and cholesterol and weight are the key impact factors in all of the LR, RF, Gradient Boosting models. The fact that we even see such cross model agreement indicates that the predictive signal is grounded in some underlying properties of the data and not a model specific artifact.

### 4.8 Explainability consistency across models

Despite differences in scanner design, the leading predictors show little variation in importance throughout the analysis. Blood pressure, age, cholesterol - these stand out across logistic regression, random forest, and Gradient Boosting alike. Notably, weight joins them as a persistent influence regardless of method used. Agreement among diverse models suggests these patterns reflect core data characteristics rather than quirks tied to any single algorithm.

Despite minor disagreements on less critical indicators like daily habit markers, these stem from how linear approaches handle relationships compared to tree-driven techniques. What happens in practice is that key predictors remain useful for clear interpretation, whereas lesser signals could benefit from deeper analysis using diverse data sources.

To quantitatively evaluate the agreement between SHAP-based feature rankings, we computed Spearman rank correlations between the feature importance rankings obtained from Logistic Regression, Random Forest, and Gradient Boosting models. The analysis shows moderate-to- strong positive correlations between model pairs, indicating that key cardiovascular risk factors such as age, systolic blood pressure, cholesterol, and weight consistently appear among the top predictors across model architectures. These results support the qualitative observations summarized in Table 3 and reinforce the concept of explainability stability.

### 4.9 Real-world applicability

The discrimination, calibration and explainability analysis indicate that the proposed framework is ready to be further developed in the form of a clinical decision-support prototype. The model’s low complexity enables inclusion in hospital information systems or telehealth platforms without unwieldy computational overhead.

Moreover, SHAP explanations give interpretable results which could assist in clinician-patient communication and probably better adherence to preventive advice. The method could thus become a user-friendly connection between predictive analytics and routine clinical healthcare processes.

### 4.10 Strengths and limitations

It is also important to comment on a few methodological and practical strengths in the present study. First and foremost, it utilizes a comprehensive public cardiovascular database having an XOR-validated DOI to enable transparency and reproducibility of the proposed approach. Second, we evaluate a range of machine learning models including linear and ensemble models using clinical dependent performance measurements (the results are thus not based on one metric or model). Third, explainable artificial intelligence approaches enable global and local interpretability which enable us to visually see the interpretability of features rather than being a black-box system. Furthermore discrimination was not only evaluated, as was often performed in previous CVD risk prediction studies, but calibration analysis and DCA were incorporated to make results more clinically meaningful.

However, there are some limitations that should be acknowledged. The analysis was limited to a cross-sectional, structured table-based data and did not contain longitudinal summaries, imaging findings or unstructured clinical notes which might improve risk prediction. In addition, despite the large and publicly available dataset used in this study no external prospective validation on independent clinical cohorts has been performed it can be an item for future research. Though limited to model-dimension, interpretation reveals clearer patterns in feature relevance - yet still lacks grounding in causality. Whether findings hold across diverse patients remains open; testing them in actual care settings demands future study. Multi-site verification alongside causal frameworks would help clarify their role down the line.

The predictive performance achieved in this study is moderate compared to some recent deep learning approaches, which reflects the study’s focus on interpretability analysis rather than optimizing predictive accuracy.

## 5 Sustainable development goal alignment

The proposed explainable machine learning framework aligns with the United Nations Sustainable Development Goal 3 (Good Health and Well-being) by supporting early and interpretable cardiovascular risk stratification using routinely available clinical variables. By combining predictive modeling with transparent SHAP-based explanations, the framework can assist clinicians in identifying high-risk individuals and promoting preventive healthcare strategies. Furthermore, the use of computationally efficient machine learning models facilitates potential deployment in resource-constrained healthcare environments, contributing to SDG 10 (Reduced Inequalities) by promoting equitable access to AI-driven healthcare tools.

## 6 Future work

Future research will focus on extending the proposed framework to more complex healthcare data environments. One promising direction is the integration of longitudinal patient records and time- series medical data, which could enable models to capture disease progression patterns over time. In addition, incorporating multimodal healthcare data—such as medical imaging, wearable sensor data, and electronic health records—may improve predictive capability while maintaining interpretability.

Another important direction involves evaluating the robustness of explainability patterns across independent clinical datasets collected from different healthcare institutions. External validation across diverse populations would help assess the generalizability of the proposed approach and strengthen its potential applicability in real-world clinical decision-support systems.

## 7 Conclusion

This study presented an explainable machine learning framework for cardiovascular disease risk prediction using structured clinical data. Three machine learning models—Logistic Regression, Random Forest, and Gradient Boosting—were evaluated using multiple classification metrics including accuracy, precision, recall, F1-score, and AUC-ROC. The results demonstrate that while predictive performance across the evaluated models was comparable, differences emerged in how each model captured interactions among clinical features.

Beyond predictive performance, the study emphasized the importance of explainable artificial intelligence for understanding model behavior in healthcare applications. SHAP-based interpretation enabled both global and local analysis of feature contributions, revealing that clinically relevant variables such as age, blood pressure, cholesterol levels, and weight consistently influenced cardiovascular risk predictions across model architectures.

The findings highlight the potential of explainable machine learning methods to improve transparency in AI-driven healthcare analytics. Rather than focusing solely on predictive accuracy, the proposed framework demonstrates how interpretability can support more reliable and accountable clinical decision-support systems based on structured patient data.

## Data Availability

The original contributions presented in the study are included in the article/supplementary material, further inquiries can be directed to the corresponding author/s.

## Footnotes

1
https://doi.org/10.21227/7qm5-dz13
